# The complete chloroplast genome of *Pseudotsuga sinensis*, a China endemic species

**DOI:** 10.1080/23802359.2022.2080012

**Published:** 2023-01-02

**Authors:** Wang Jun Li, Tu Feng, Jun Li, Bin He, Shun Zou, Peng Ju Liu

**Affiliations:** a Guizhou Province Key Laboratory of Ecological Protection and Restoration of Typical Plateau Wetlands, Guizhou University of Engineering Science, Bijie, China; b Nature Reserve Service Station for Pseudotsuga sinensis in Weining, Bijie, China; c CAS Key Laboratory for Plant Diversity and Biogeography of East Asia, Kunming Institute of Botany, Chinese Academy of Sciences, Kunming, China

**Keywords:** *Pseudotsuga sinensis*, chloroplast genome, phylogeny

## Abstract

In this study, we collected plant material from *Pseudotsuga* *sinensis* in Guizhou, China, and sequenced it. The complete chloroplast genome consisted of 122,243 bp, including a large single-copy (LSC) region, a small single-copy (SSC) region, and two inverted repeat regions like those in *P. sinensis* var. *wilsoniana*. The GC content of *P. sinensis* and *P. sinensis* var. *wilsoniana* are 38.7% and 38.8%, respectively. The reconstructed phylogenetic tree reveals that *P. sinensis* was a sister species to *P. sinensis* var. *wilsoniana*. Hence, the availability of the chloroplast genome of *P. sinensis* will promote further phylogenetic studies of the family Pinaceae.

*Pseudotsuga* is a small genus of evergreen coniferous trees with four or five recognized species that are discontinuously distributed across East Asia and North America. It also is a rare relict plant unique to China and is classified as ‘endangered’ by the International Union for Conservation of Nature (IUCN; Li et al. 2021). Prior to this study, the NCBI database only contained chloroplast genomes for two *Pseudotsuga* species. The chloroplast genome size of most reported pinaceous species only ranges from 107 to 120 kb (Lin et al. 2010; Wu et al. 2011). To better address evolution-based questions regarding *Pseudotsuga*, we assembled and reported the first complete plastome of *Pseudotsuga sinensis* (Dode 1912).

Plant material from *P. sinensis* was collected from a village in Guizhou Province (26°35′11.04″N, 103°58′5.88″E). Voucher specimens (LPJ001) were deposited in the Herbarium of the Kunming Institute of Botany at the Chinese Academy of Sciences (KUN, 1519936, http://www.kun.ac.cn/). Total genomic DNA was collected from fresh leaves of an *Aralia elata* sample based on a previously suggested method (Li et al. 2013). Next, a shotgun library was constructed, and high-throughput sequencing was conducted on an Illumina Hiseq X-Ten sequencing platform. The clean data were assembled *de novo* using GetOrganelle v1.7.5.0 (Jin et al. 2020), and annotation was performed using GeSeq (Tillich et al. 2017). Subsequently, the annotations of *P. sinensis* were inspected manually using Geneious v9.0.2 (Kearse et al. 2012) and compared with *P. sinensis* var. *wilsoniana*. Finally, the annotated genomic sequence of *P. sinensis* was submitted to GenBank with the accession number MZ779058.

The complete chloroplast genome *P. sinensis* consists of 122,243 bp, including a large single-copy (LSC) region, a small single-copy (SSC) region, and two inverted repeating regions like those in *P. sinensis* var. *wilsoniana*. The GC content of *P. sinensis* is 38.7%, and it has a 22,332-bp inverted repeat region compared to the complete chloroplast genome of *P. sinensis* var. *wilsoniana*. The complete chloroplast genome of *P. sinensis* var. *wilsoniana* includes 111 genes, including 71 protein-coding sequences, 36 tRNA-encoding genes, and 4 rRNA-encoding genes. The complete chloroplast genome of *P. sinensis* var. *wilsoniana* includes 113 genes, including 73 protein-coding sequences, 36 tRNA-encoding genes, and 4 rRNA-encoding genes. The complete chloroplast genome of *P. sinensis* lacks the genes *psbN* and *ycf12*.

To obtain insight into the position of *P. sinensis* within the family Pinaceae, 19 other complete chloroplast genome sequences of Pinaceae were downloaded from the GenBank database, using two sequences of *Cunninghamia lanceolata* as outgroups (Figure 1). Phylogenetic analyses were performed using maximum-likelihood (ML) in RAxML8.0 with 1000 bootstrap replicates (Stamatakis 2014). In the ML tree, most of the nodes had a 100% bootstrap value. The reconstructed phylogenetic tree revealed that *P. sinensis* is a sister species to *Pseudotsuga brevifolia* and *P. sinensis* var. *wilsoniana*. Although morphological data suggest that *P. sinensis* is more closely related to *P. sinensis* var. *wilsoniana* than to *P. brevifolia*, our analysis indicates close relationships between all three species. The ML tree based on complete chloroplast genome data did not provide further insights into the relationship between the three species. However, the addition of the whole chloroplast genome of *P. sinensis* to the NCBI database will aid in the phylogenetic reconstruction of the family Pinaceae.

**Figure 1.**
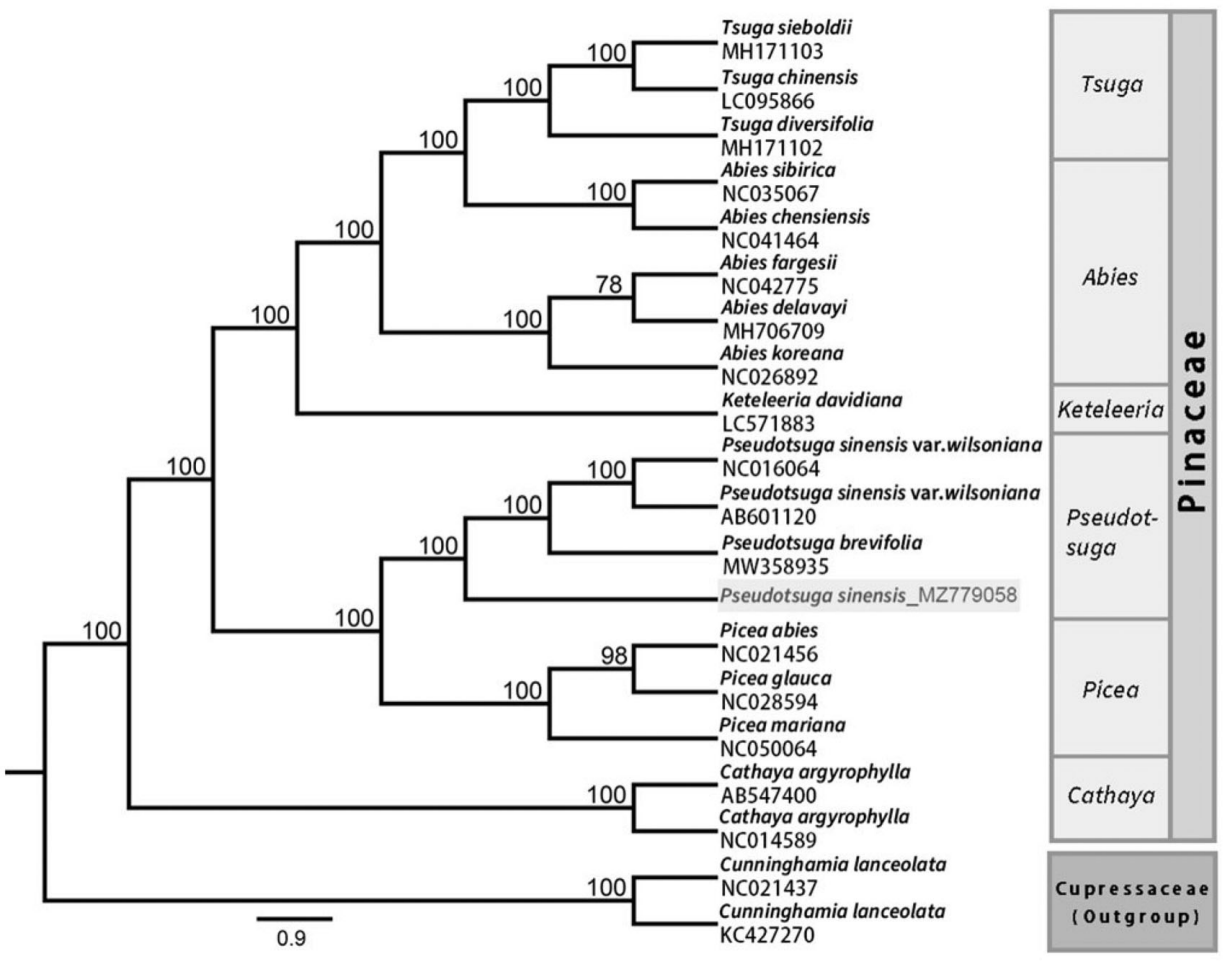
Maximum-likelihood phylogenetic tree based on the chloroplast genome sequences. Numbers at nodes represent bootstrap support values.

## Ethical approval

Fieldwork was approved and supervised by the Nature reserve service station for *Pseudotsuga sinensis* in Weining. *Pseudotsuga sinensis* is vulnerable (VU) according to IUCN Red list, so all the plant experiments were conducted according to policies research involving species at risk of extinction described in ‘the International Union for Conservation of Nature.’

## Data Availability

The genome sequence data that support the findings of this study are openly available in GenBank of NCBI at [https://dataview.ncbi.nlm.nih.gov/] under accession no. MZ779058. The associated BioProject, SRA, and Bio-Sample numbers are PRJNA755471, SRR15497848, and SAMN20824202 respectively.
